# Crimean-Congo Hemorrhagic Fever in Bulgaria

**DOI:** 10.3201/eid1008.040162

**Published:** 2004-08

**Authors:** Anna Papa, Iva Christova, Evangelia Papadimitriou, Antonis Antoniadis

**Affiliations:** * WHO Collaborating Center for Reference and Research on Arboviruses and Haemorrhagic Fever Viruses at Aristotle University of Thessaloniki, Thessaloniki, Greece;; † National Center of Infectious and Parasitic Diseases, Sofia, Bulgaria

**Keywords:** Crimean Congo Hemorrhagic Fever, Bulgaria, dispatch

## Abstract

We report the epidemiologic characteristics of Crimean-Congo hemorrhagic fever in Bulgaria, as well as the first genetic characterization of the virus strains circulating in the country in 2002 to 2003 that caused disease in humans.

Crimean-Congo hemorrhagic fever virus (CCHFV) (genus *Nairovirus*, family *Bunyaviridae*) causes severe disease with a fatality rate as high as 30%. CCHFV is endemic in the Balkan Peninsula; a number of cases occur every year, sometimes in an epidemic form. Cases have been reported in Albania (1), Kosovo (2,3), and Bulgaria (4). Mountains approximately 1,500 m to 2,500 m high separate these countries from Greece, where no case of the disease has yet been identified. However, a CCHFV strain was isolated in Greece from *Rhipicephalus bursa* ticks, collected in May 1975 from goats of a flock in Vergina village, 80 km west of Thessaloniki (5). Antibodies against the virus were detected in the Greek human population (6). CCHFV is also endemic in Russia and in parts of Asia and Africa.

The virus is transmitted to humans by the bite of ixodid ticks (primarily of the *Hyalomma* genus) or by contact with blood or tissues from infected persons or infected livestock. The risk for spread of the virus from person to person is high, which occasionally results in nosocomial outbreaks. After an incubation period of 3 to 7 days, the patient has sudden onset of fever, chills, myalgia, and headache, which rapidly progress to severe illness; a hemorrhagic state follows with bleeding from the mucous membranes and petechiae, associated with thrombocytopenia and leukopenia (7).

CCHFV, like all members of the genus, is a negative-stranded RNA virus with a tripartite genome consisting of a small, medium, and large segment encoding the nucleocapsid protein; the glycoprotein precursor, which results in the two envelope glycoproteins G1 and G2; and the putative RNA-dependent polymerase, respectively (8).

We report (for the first time in English) the epidemiologic characteristics of the disease in Bulgaria. We also provide the first genetic characterization of the CCHFV strains circulating in the country from 2002 to 2003 that caused disease in humans.

Bulgaria is a country of 8 million inhabitants in the eastern part of the Balkans (Figure 1). CCHF was first recognized in the country in 1952 and became a reportable disease in 1953. In 1968, CCHFV was isolated from blood samples of two patients. Results from serologic investigations showed that approximately 20% of patients living in disease-endemic areas who reported a tick bite had antibodies to CCHFV (4). The seropositivity in animals in the disease-endemic areas can be as high as 50%. Most cases were reported from Plovdiv and Pazardgik (central Bulgaria), Haskovo and Kardgali (southeastern Bulgaria), Shumen (northeastern Bulgaria), and Burgass (eastern Bulgaria) (4). The most prevalent tick in Bulgaria is *Ixodes ricinus*; however CCHFV strains have been isolated from *Hyalomma plumbeum* (*H. marginatum*), *Rhipicephalus sanguineus*, and *Boophilus calcaratus* (9).

**Figure 1:**
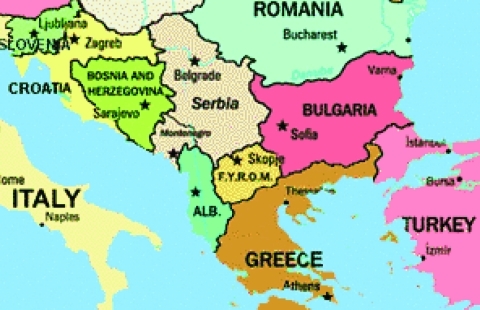
Bulgaria and neighboring Balkan countries.

From 1953 to 1974, 1,105 CCHFV cases were reported to the Bulgarian Ministry of Health; the fatality rate was approximately 17%. Of them, 20 cases were nosocomial infections and 52% were fatal. In 1974, an immunization program was introduced for medical workers and military personnel in CCHF-endemic areas. The treatment regimen consisted of mouse brain preparation inactivated by chloroform, heated at 58°C, and adsorbed on Al(OH)_3_. The first two doses were given at day 0 and day 30; a third dose was given 1 year later, and another dose was given 5 years after that (10). As a result, between 1975 and 1996, the number of reported CCHF cases was reduced to 279, with a fatality rate of 11.4%. No infection was reported from vaccinated military personnel (11).

Since 1997, a total of 124 cases occurred in Bulgaria, 27 of them fatal (Table 1). Most patients had been bitten by a tick; however, a few were infected through direct contact with CCHF patients. Only the eastern part of the country has been affected; two main foci exist, one in the southeast and a second one in northeast. The mean age of patients is 52 years (range 11–79 years). Most patients are men (74%), probably because they are more frequently exposed to ticks bites during outdoor activities. The disease occurs mainly from March to July when ticks are more active. The main clinical symptoms are fever, malaise, nausea, epistaxis, petechiae, and bleeding from the gastrointestinal tract; the main laboratory findings are leukopenia, thrombocytopenia, and elevated transaminase levels.

**Table 1:** Distribution of Crimean-Congo hemorrhagic fever cases and related deaths in Bulgaria, 1997–2003

Y	No. of cases	No. of deaths (%)
1997	20	4 (20)
1998	15	3 (20)
1999	5	2 (40)
2000	10	1 (10)
2001	18	5 (28)
2002	56	12 (21)
2003	14	2 (14)

To investigate the genetic relationships of the CCHFV strains circulating recently in Bulgaria, RNA was extracted from cell culture supernatant from six virus isolates. The virus had been isolated in a Vero E6 cell line from blood samples taken from CCHF patients who were infected in 2002 and 2003. The epidemiologic characteristics of the patients are shown at Table 2. A reverse-transcriptase–nested polymerase chain reaction (PCR) was applied to amplify a partial fragment of the S RNA genome segment by using two sets of primers, F2-R3 and F3-R2 (12). Purified PCR products were sequenced; the nucleotide sequences were submitted to the GenBank database and assigned the accession numbers AY550253–AY550258. After aligning the obtained Bulgarian CCHFV sequences with respective ones retrieved from GenBank, we constructed a phylogenetic tree with PHYLIP software (13) (Figure 2). All Bulgarian isolates were found to cluster together, with a genetic homology of 98.4% to 100% at the nucleotide level. Identical sequences were obtained from isolates originating from the same region in the same year.

**Table 2:** Epidemiologic characteristics of patients whose blood samples yielded Crimean-Congo hemorrhagic fever virus strains

No. of isolate	Date of disease onset	Sex	Age (y)	Area
BUL1/03	June 2003	Male	60	Haskovo
BUL2/03	June 2003	Male	45	Shumen
BUL3/02	April 2002	Female	62	Shumen
BUL6/02	June 2002	Male	73	Plovdiv
BUL9/02	June 2002	Male	23	Shumen
BUL10/02	August 2002	Male	39	Haskovo

**Figure 2:**
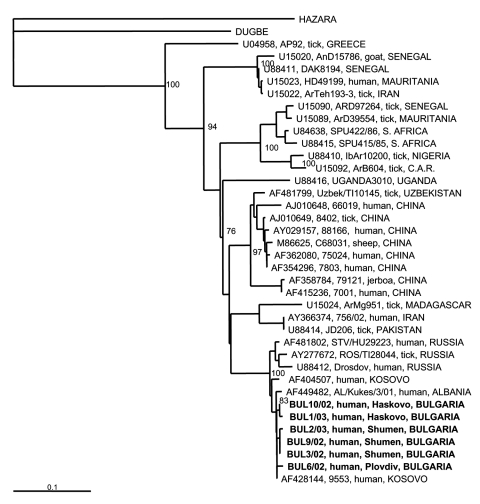
Phylogenetic tree based on 255-nt fragment from the S RNA segment, showing the clustering of the sequences obtained from this study and respective representative Crimean-Congo hemorrhagic fever virus strains from GenBank database. Sequences of two other nairoviruses, Dugbe and Hazara, were included; Hazara virus was used as outgroup. The numbers indicate percentage bootstrap replicates (of 100); values below 70% are not shown. Horizontal distances are proportional to the nucleotide differences. The scale bar indicates 10% nucleotide sequence divergence. Vertical distances are for clarity only. Sequences used in the analysis are indicated at the tree as: GenBank accession no., strain, host, country. C.A.R., Central African Republic.

The Bulgarian CCHFV strains were found to cluster with other Balkan strains from Kosovo and Albania, with a mean genetic difference of 2% and 1.2%, respectively. All Balkan strains clustered in the same branch with CCHFV strains from European Russia, such as STV/HU29223 strain, isolated in 2000 from human blood in Stavropol (mean genetic difference 2.5%), and ROS/TI28044, isolated in 2000 from *Hyalomma marginatum* ticks in Rostov (mean genetic difference 3.7%) (14). A "European CCHFV group," distinct from all others, is evident. An exception to the European group is the Greek strain AP92, isolated from *R. bursa* ticks (5), which forms an independent clade, which differs from the Bulgarian strains by 24%. This genetic difference is likely attributable to the different species of related ticks or to reassortment. Studies on the Greek strain are still in progress; they will help explain the genetic and pathogenic differences among this strain and respective strains from neighboring countries.

Although the genetic divergence among European strains is low, a great divergence is seen among European CCHFV strains and strains from other continents (Asia and Africa). As the number of CCHFV sequences derived from the S genome segment is growing, eight distinct clades can be seen: 1) strain AP92 from Greece; 2) strains from Senegal, Mauritania, and Iran; 3) strains from Senegal, Mauritania, and South Africa; 4) strains from Nigeria and Central African Republic; 5) strain from Uganda; 6) strains from Central Asia and China; 7) strains from Madagascar, Iran, and Pakistan; and 8) European strains (Russia, Albania, Kosovo, and Bulgaria) (Figure 2).

In conclusion, this report shows that the CCHV is endemic in Bulgaria and causes severe disease in the whole Balkan Peninsula (except Greece) and that the Bulgarian CCHFV strains are genetically similar to other Balkan virus strains (except AP92). CCHFV evolves relatively slowly, which suggests that the great genetic divergence among the strains is not time-dependent. Whether this divergence is because of the different tick species, the different geographic location, or any other reason, remains to be elucidated.
